# Landscape analysis of m6A modification regulators related biological functions and immune characteristics in myasthenia gravis

**DOI:** 10.1186/s12967-023-03947-5

**Published:** 2023-03-02

**Authors:** Shuang Li, Hui Liu, Zhe Ruan, Rongjing Guo, Chao Sun, Yonglan Tang, Xiaoxi Huang, Ting Gao, Sijia Hao, Huanhuan Li, Na Song, Yue Su, Fan Ning, Zhuyi Li, Ting Chang

**Affiliations:** 1 Department of Neurology, Tangdu Hospital, The Fourth Military Medical University, Xi’an, 710038 Shaanxi China; 2 Xi’an Medical University, Xi’an, 710021 Shaanxi China

**Keywords:** Myasthenia gravis, m6A modification, Immune characteristics, Correlation analysis, WGCNA

## Abstract

**Background:**

N6-methyladenosine (m6A) modification has been recognized to play fundamental roles in the development of autoimmune diseases. However, the implication of m6A modification in myasthenia gravis (MG) remains largely unknown. Thus, we aimed to systematically explore the potential functions and related immune characteristics of m6A regulators in MG.

**Methods:**

The GSE85452 dataset with MG and healthy samples was downloaded from Gene Expression Omnibus (GEO) database. m6A modification regulators were manually curated. The targets of m6A regulators were obtained from m6A2Target database. The differential expressed m6A regulators in GSE85452 dataset were identified by “limma” package and were validated by RT-PCR. Function enrichment analysis of dysregulated m6A regulators was performed using “clusterProfiler” package. Correlation analysis was applied for analyzing the relationships between m6A regulators and immune characteristics. Unsupervised clustering analysis was used to identify distinct m6A modification subtypes. The differences between subtypes were analyzed, including the expression level of all genes and the enrichment degree of immune characteristics. Weighted gene co-expression network analysis (WGCNA) was conducted to obtain modules associated with m6A modification subtypes.

**Results:**

We found that CBLL1, RBM15 and YTHDF1 were upregulated in MG samples of GSE85452 dataset, and the results were verified by RT-PCR in blood samples from19 MG patients and 19 controls. The targeted genes common modified by CBLL1, RBM15, and YTHDF1 were mainly enriched in histone modification and Wnt signaling pathway. Correlation analysis showed that three dysregulated m6A regulators were closely associated with immune characteristics. Among them, RBM15 possessed the strongest correlation with immune characteristics, including CD56dim natural killer cell (r = 0.77, P = 0.0023), T follicular helper cell (r = − 0.86, P = 0.0002), Interferon Receptor (r = 0.78, P = 0.0017), and HLA-DOA (r = 0.64, P = 0.0200). Further two distinct m6A modification patterns mediated by three dysregulated m6A regulators was identified. Bioinformatics analysis found that there were 3029 differentially expressed genes and different immune characteristics between two m6A modification patterns. Finally, WGCNA analysis obtained a total of 12 modules and yellow module was the most positively correlated to subtype-2.

**Conclusion:**

Our findings suggested that m6A RNA modification had an important effect on immunity molecular mechanism of MG and provided a new perspective into understanding the pathogenesis of MG.

**Supplementary Information:**

The online version contains supplementary material available at 10.1186/s12967-023-03947-5.

## Introduction

Myasthenia gravis (MG) is an autoimmune disease of nervous system mainly caused by the autoantibodies against acetylcholine receptor (AChR) in the postsynaptic membranes of neuromuscular junction [1]. Other antibodies against muscle-specific kinase (MuSK), low-density lipoprotein receptor-related protein 4 (LRP4), and Agrin are also detectable in a few MG patients [2]. MG is a complex multifactorial disease, the development of which involves environment, genetic predisposition, and immunity. In the past 5 years, the explorations on immune dysregulations in MG have extended. Some circulating molecules, such as IL17, IL21, BAFF, and miRNAs have been reported to be biomarkers for MG [3]. The dysregulations of these molecules may reflect the abnormity in the proportions or activation states of immune cell subpopulations [4]. A study has claimed that the proportion of thymic and circulating T follicular helper cells increases in MG, which may further contribute to the development of thymic germinal center, but also the activation of B-cell and the production of antibodies [5]. Immune dysregulation is clear mark of autoimmunity in MG, but the exact regulation factors remain indistinct.

Epigenetics, the bridges of the interaction between environmental factors and genetic factors, refer to heritable modification that regulate gene expression without nucleotide sequence alterations. N6-methyladenosine (m6A) modification, one of the most prevalent RNA modifications in epigenetic mechanisms, has been proved to play an important part in a variety of biological processes [6, 7]. m6A modification is a dynamic and reversible process, which depends on the functions of m6A regulators including methyltransferases (writers), demethylases (erasers), and binding proteins (readers) [8]. Accumulating evidence suggests that m6A modification is required for the development, differentiation, activation, migration and polarization of immune cells, thus regulating immune response. For example, Wang et al. showed that METTL3-mediated mRNA methylation enhanced the translation of CD40, CD80, TLR4 signaling adaptor Tirap transcripts, furtherly promoting the maturation and function of dendritic cell (DC) and DC-based T cell response [9]. The roles of immune cells in the pathogenesis of autoimmune diseases have been widely investigated, and these cells have been proved to participate in the development of autoimmune disorders. Some studies have reported that m6A modification can affect the progression of autoimmune diseases by regulating immune cells. T cell-specific ablation of m6A eraser ALKBH5 conferred protection against experimental autoimmune encephalomyelitis (EAE). Mechanistically, the deficiency of ALKBH5 increased the m6A modification level of the CXCL2 and IFN-γ mRNAs and subsequently decreased their transcript stability and protein expression during the induced neuroinflammation, furtherly attenuating CD4 + T cell-mediated responses and inflammatory cell infiltration in the central nervous system [10]. Furthermore, the important impacts of m6A modifications on systemic lupus erythematosus [11], multiple sclerosis [12], rheumatoid arthritis [13], and autoimmune thyroid disease [14] have also been studied. These studies indicated that m6A RNA modifications were closely associated with the development of autoimmune diseases.

Here, we systematically investigated the biological functions and immune characteristics of m6A epigenetic regulation in MG samples. We then performed unsupervised clustering analysis based on the expression levels of m6A regulators and identified two distinct m6A modification patterns in MG. Furtherly, GO-BP enrichment analysis of differentially expressed genes between two patterns was employed and WGCNA analysis was applied to identify key module related to modification patterns. As far as we know, our work, for the first time, comprehensively analyzed m6A’s roles in MG, which might provide a novel view to identify biomarkers for MG treatment and diagnosis.

## Materials and methods

### Data acquisition and processing

The dataset of mRNA expression profile under accession number GSE85452 [15], which included 13 MG samples and 12 healthy samples, was downloaded from Gene Expression Omnibus (GEO) database (https://www.ncbi.nlm.nih.gov/geo/) by “GEOquery” R package. The mRNA expression profile of CD14 monocytes was performed on peripheral blood of all samples using Illumina HumanHT-12 V4.0 expression beadchip. Gene probes were annotated as gene symbols based on platform annotation file. Gene probes, having multiple matching gene symbols or without matching gene symbols, were excluded. The median value was selected as the expression value of duplicate gene symbols.

We manually curated m6A RNA methylation modification regulators through reviewing related publications. A gene list of m6A modification regulators was obtained, including 8 writers (METTL3, METTL14, WTAP, KIAA1429, RBM15, RBM15B, CBLL1, and ZC3H13), 2 erasers (FTO and ALKBH5) and 13 readers (YTHDF1, YTHDF2, YTHDF3, YTHDC1, YTHDC2, HNRNPC, HNRNPA2B1, IGF2BP1, IGF2BP2, IGF2BP3, FMR1, ELAVL1, and LRPPRC).

### Differentially expression analysis and screening MG-related m6A regulators

The differentially expression genes (DEGs) between MG samples and healthy samples were determined using “Empirical Bayes method” in the R package “limma”. The cutoff value of |Fold Change (FC)|> 1 and P-value < 0.05 were considered as statistically significant. Then, m6A modification regulators were mapped into DEGs, and the overlapped genes were the differentially expressed m6A regulator genes (DEMRGs) and defined as MG-related m6A regulators. Subsequently, volcano plot and heatmap were draw to visualize the DEGs and DEMRGs between MG and healthy samples, respectively. Box plot were draw to present the expression status of all m6A regulators between two groups.

### Functional annotation of MG-related m6A regulators

According to the GSE85452 dataset, Pearson correlation analysis was performed with R to evaluate the expression correlation of m6A modification regulators in MG samples and healthy samples, and further identify the co-expressed genes of DEMRGs in MG samples. The genes with |r|> 0.5 and P < 0.05 were selected as significant co-expressed genes. Meanwhile, we predicted the target genes of DEMRGs using m6A2Target database (http://m6a2target.canceromics.org) [16]. The intersection genes between co-expression and predicted targets were defined as target genes modified by MG-related m6A regulators. Gene Ontology biological process (GO-BP) enrichment analysis and Kyoto Encyclopedia of Genes and Genomes (KEGG) pathway analysis were employed using R package “clusterProfler” to explore the biological functions of dysregulated m6A regulators according to the target genes. P-value < 0.05 was regarded as significant GO-BPs and pathways.

### Correlation analysis between m6A regulators and immune characteristics

In this study, we analyzed the relationships between MG-related m6A regulators and immune characteristics including immune cell subpopulations, immune reaction pathways and HLA genes. Single-sample gene-set enrichment analysis (ssGSEA) was applied to assess the score of the 28 types of immune cell subpopulations and 17 immune reaction pathways in MG samples. According to a previous study, the gene sets used to evaluate the enrichment degree of immune cell subpopulations were obtained [17]. Meanwhile, the gene sets related to immune reaction pathways were downloaded from ImmPort database (http://www.immport.org) [18], and the expression level of HLA genes was from GSE85452 dataset. The correlations of m6A regulators with immunocyte fractions, immune reaction pathway and HLA gene expression were calculated using Pearson correlation analysis. |r|> 0.5 was considered as strongly correlated and P-value < 0.05 was statistically significant.

### Identification of m6A regulators related modification patterns

To identify different m6A regulators related modification patterns, we performed unsupervised clustering analysis based on the MG-related m6A regulators. The consensus clustering algorithm was applied to estimate the cluster numbers and robustness [19]. The robustness of classification was guaranteed using the R package “ConsensuClusterPlus” with the above steps for 1000 iterations. Principal component analysis (PCA) was further employed to validate the expression status of MG-related m6A regulators in different modification patterns. Further, the expression status of all m6A regulators in two modification patterns were evaluated by t test. P-value < 0.05 was considered as statistically significant.

### Comprehensive features analysis of distinct modification patterns

Distinct modification patterns might present different immune status in the molecular mechanism. Thus, we also compared the differences of immune cell subpopulations, immune reaction pathways and the expression levels of HLA genes between two subtypes using t test. In addition, to identify the differences of all genes between two distinct modification patterns, we conducted differential expression analysis by the R package “limma”. Genes with |FC|> 1 and P-value < 0.05 were considered as differential genes between two modification patterns.

### Biological functions and related modules analysis of two m6A modification patterns

To explore the biological functions of m6A modification patterns, GO-BP enrichment analysis was performed on differentially expressed genes between two distinct subtypes. To identify the significant modules related different modification patterns, weighted gene co-expression network analysis (WGCNA) was then conducted by the “WGCNA” package in R [20]. We employed the following major steps: (1) checking if any obvious outliers were existed based on cluster of all samples; (2) constructing a co-expression network using the matrix of pairwise Pearson correlation coefficients; (3) clustering genes into different functional modules with different colors according to the dynamic tree cut algorithm with min; (4) calculating module membership (MM) and gene significance (GS) to correlate modules with phenotype; (5) extracting the key module gene information was used for further analysis.

### Clinical samples

In total, 19 MG patients were recruited for the sampling of peripheral blood from the Department of Neurology at Tang Du Hospital from September 2022 to December 2022. The detailed clinical information of MG group was showed in Additional file 1: Table S1. Meanwhile, 19 healthy controls, 9 males and 10 females aged 29–73 years (mean, 55.0 ± 14.9 years), were selected among volunteers. Peripheral blood samples collected from of subjects were put in tubes containing ethylenediaminetetraacetic acid. Peripheral blood mononuclear cells (PBMCs) were further isolated using lymphocyte separation. Both MG patients and healthy controls signed a consent form for the collection of data and blood samples. The study was approved by the local ethics committee.

### RT-PCR analysis

Total RNA was isolated with TRIzol reagent (Invitrogen, USA) according to the manufacturer’s instructions. The extracted RNA was reverse transcribed into cDNA using a *Evo M-MLV* RT Kit with gDNA Clean for qPCR II (Accurate Biotechnology, Human, China). Quantitative real-time PCR analysis was performed using 2xSYBR Green Premix Pro Taq HS qPCR Kit (Accurate Biotechnology, Human, China). The 2^−ΔΔCt^ method was used for the calculation of relative expression level normalized by GAPDH.

### Statistical analysis

GraphPad Prism version 6.0 was used to perform the statistical analysis. Data was presented as the mean ± standard deviation (SD). Student’s t test was performed to analyze the differences between the two groups. P-value < 0.05 (*) was considered statistically significant.

## Results

### Identification of MG-related m6A modification regulators

**Fig. 1:**
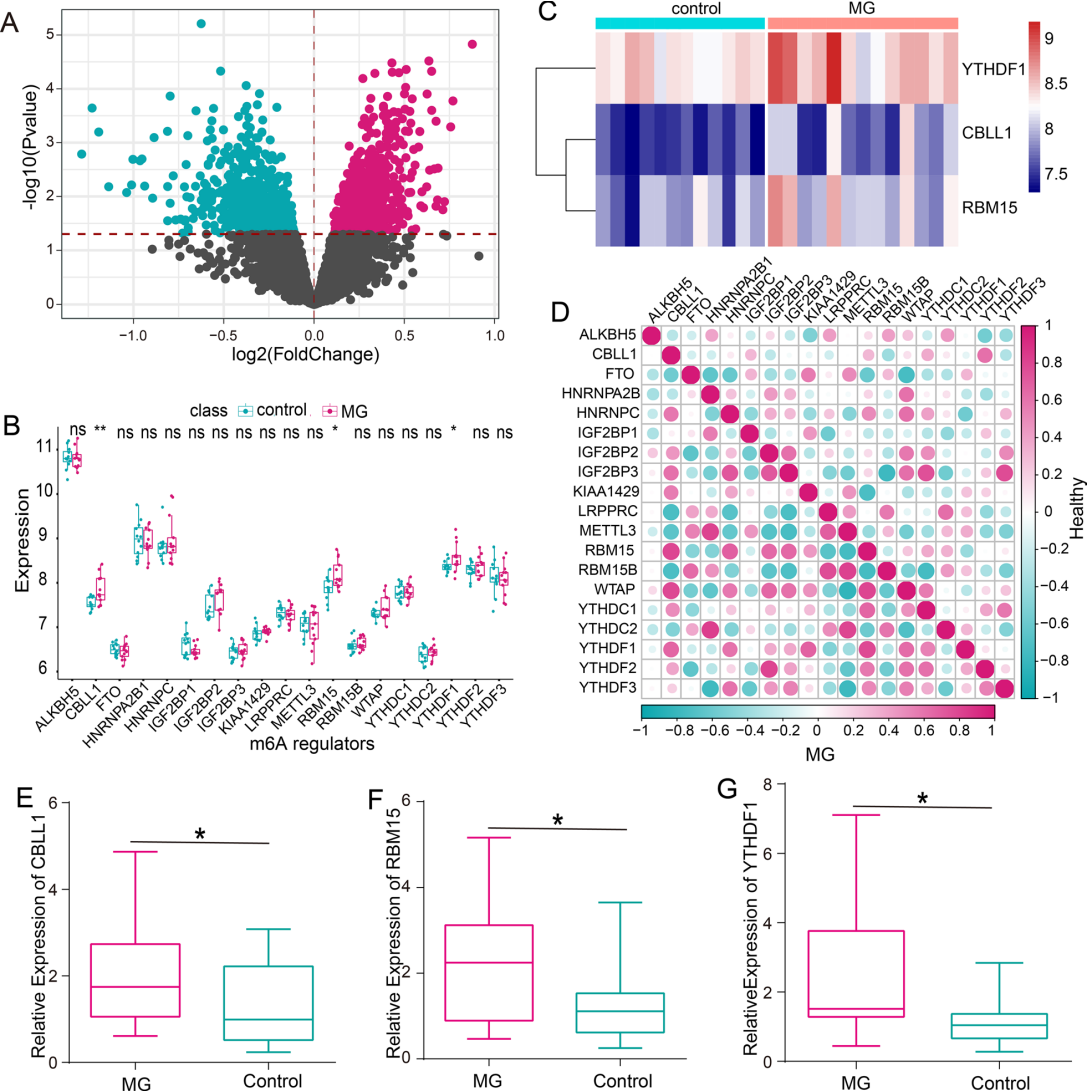
Expression landscape of m6A regulators in MG. **A** Volcano plot of differentially expressed genes between MG samples and healthy samples. **B** Box plot of the expression status of 19 m6A regulators. **C** Heatmap of MG-related dysregulated m6A regulators. **D** Comparison of the correlations among all m6A regulators between MG samples and healthy samples. **E** The expression of CBLL1 was examined in MG patients and control subjects by RT-PCR. **F** The expression of RBM15 was examined in MG patients and control subjects by RT-PCR. **G** The expression of YTHDF1 was examined in MG patients and control subjects by RT-PCR. *p < 0.05

In this study, we compared the expression status of all genes between MG samples and healthy samples, and the DEGs were identified and shown in the volcano plot (Fig. 1A). We manually acquired 23 m6A RNA modification regulators, but only 19 regulators were expressed in CD14 monocytes (Fig. 1B). Among them, we observed that CBLL1, RBM15, and YTHDF1 were overexpressed in MG samples than that in healthy samples (*P* < 0.05) (Fig. 1C). In addition, there were no obvious differences between MG and healthy samples with regard to ALKBH5, FTO, HNRNPA2B1, HNRNPC, IGF2BP1, IGF2BP2, IGF2BP3, KIAA1429, LRPPRC, METTL3, RBM15B, WTAP, YTHDC1, YTHDC2, YTHDF2, and YTHDF3 expression levels. To evaluate whether m6A regulators functioned crucially in the development of MG, we analyzed the correlation among them in MG samples and healthy samples, separately. The results indicated that the correlations among m6A regulators had significant changes between MG and healthy samples (Fig. 1D). Interestingly, the correlations among CBLL1, RBM15, and YTHDF1 were stronger in MG samples than that in healthy samples. Meanwhile, we collected PBMCs with MG patients to detect the expression levels of CBLL1, RBM15 and YTHDF1. The result of RT-PCR showed that CBLL1, RBM15 and YTHDF1 were upregulated in MG patients than healthy controls (Fig. 1E–G).

### Potential functions of targeted genes of MG-related modification regulators

**Fig. 2:**
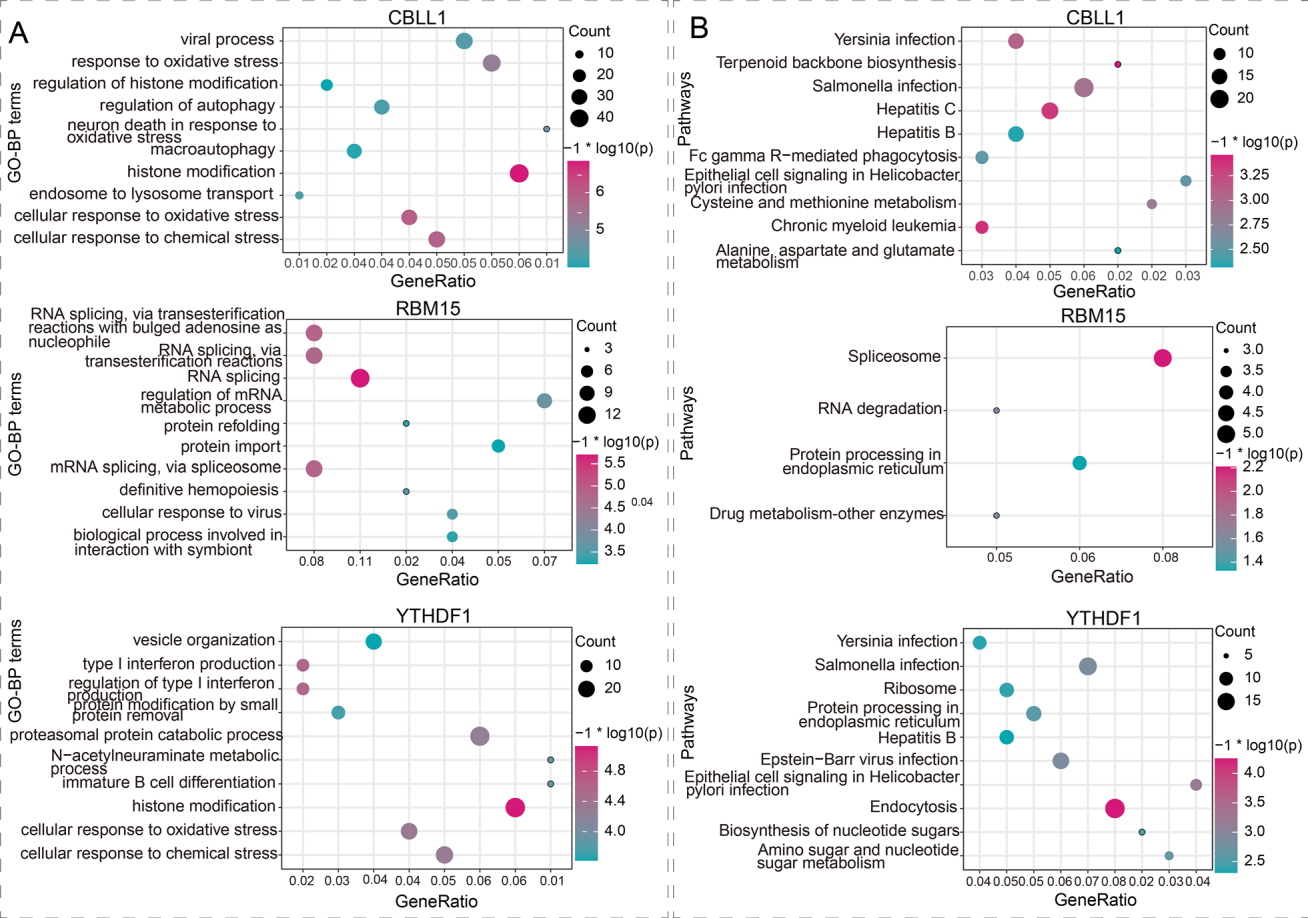
Function enrichment analysis of CBLL1, RBM15, and YTHDF1, respectively. **A** Top 10 GO-BP terms enriched by the targets of CBLL1, RBM15, and YTHDF1, respectively. The degree of enrichment increases from green to red. **B** Top 10 pathways enriched by the targets of CBLL1, RBM15, and YTHDF1, respectively. The degree of enrichment increases from green to red

To explore the potential functions of m6A regulators in MG, a series of bioinformatics analyses were performed. The results of co-expression analysis demonstrated that CBLL1 expression was significant correlated with 2381 genes, and RBM15 and YTHDF1 expression levels had a dramatically co-expression relationship with 2219 and 1028 genes, respectively. In addition, the genes possibly modified by CBLL1, RBM15, and YTHDF1 from m6A2Target database were shown in Additional file 2: Table S2. Ultimately, a total of 819, 138, and 531 were separately regulated by CBLL1, RBM15, and YTHDF1 through m6A RNA modification in MG samples (|r|> 0.5 and P < 0.05) (Additional file 3: Table S3). Furtherly, GO and KEGG enrichment analysis were performed based on the genes regulated by CBLL1, RBM15, and YTHDF1, respectively. As a result, we observed a total of 565, 189, 405 GO-BP terms (Additional file 4: Table S4) and 40, 4, 29 pathways (Additional file 5: Table S5) significantly enriched by CBLL1, RBM15, YTHDF1, respectively. The findings showed that CBLL1 was mainly involved in histone modification and response to oxidative stress related GO-BP terms, while RBM15 was in RNA splicing related GO-BP terms and YTHDF1 was in histone modification and type I interferon production related GO-BP terms (Fig. 2A). The KEGG results indicated that CBLL1 was correlated with chronic myeloid leukemia, hepatitis C, cysteine and methionine metabolism, et al., while RBM15 was with spliceosome pathway and YTHDF1 was with endocytosis pathway (Fig. 2B).

**Fig. 3:**
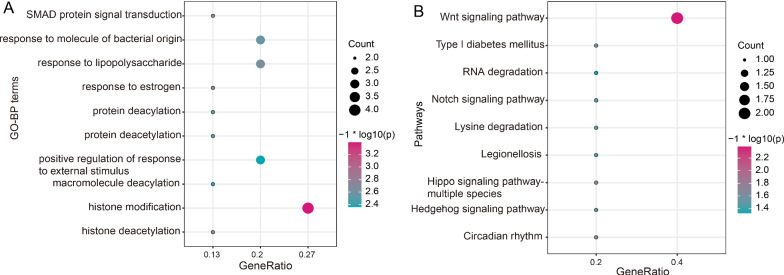
The potential functions of CBLL1, RBM15, and YTHDF1 based on common targets. **A** Top 10 GO-BP terms. **B** Top 10 pathways

We also predicted the potential functions of these three m6A regulators according to the genes common modified by CBLL1, RBM15, and YTHDF1. We observed that they mainly participated in histone modification (Fig. 3A) and Wnt signaling pathway (Fig. 3B).

### The correlations between m6A modification regulators and immune characteristics in MG

**Fig. 4:**
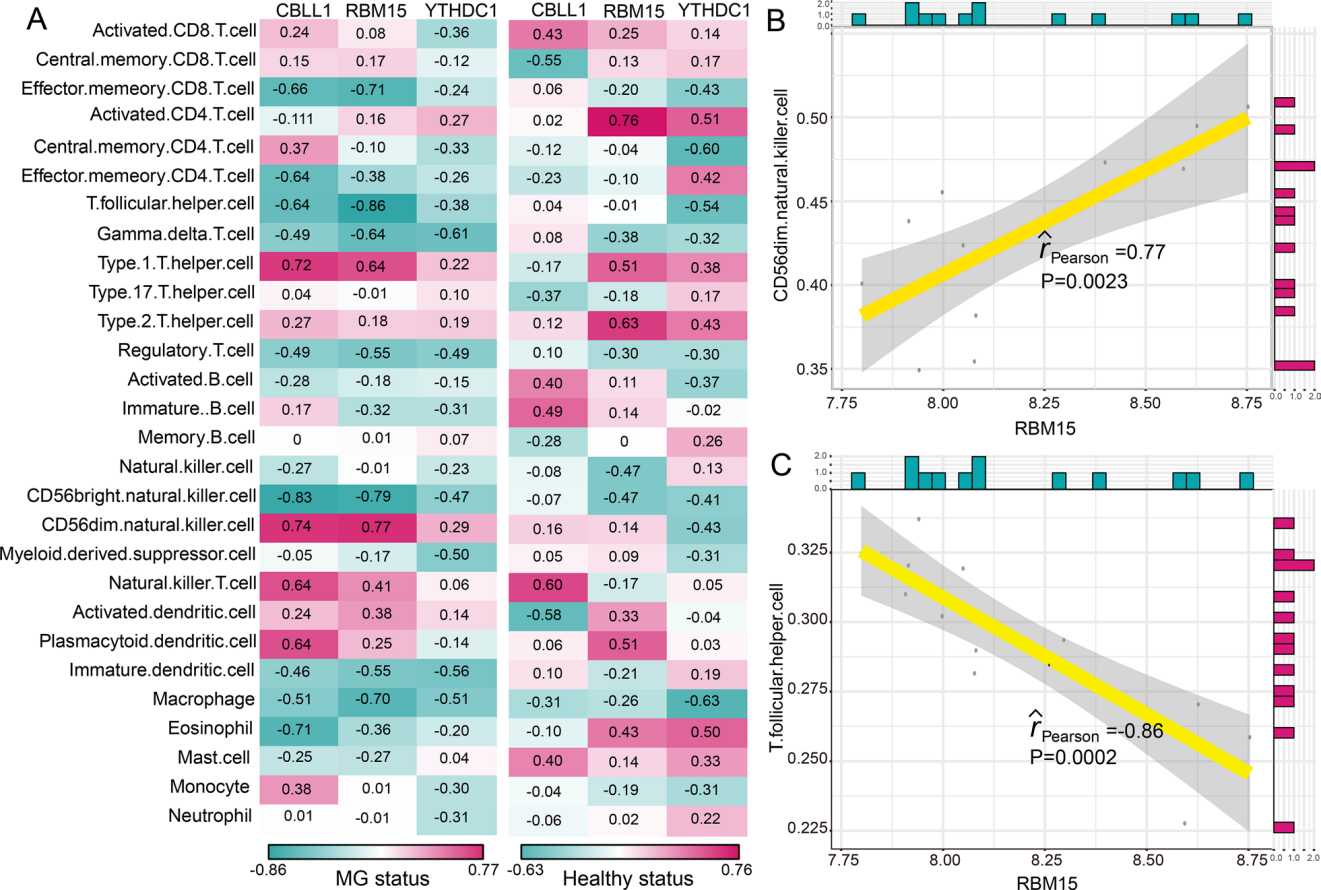
The correlations between immune cell subpopulations and dysregulated m6A regulators. **A** The dot-plot showed the correlations between each immunocyte cell type and each dysregulated m6A regulators in MG samples and healthy samples. The positive correlation became stronger as the red color darkened, and the negative correlation became stronger as the green color darkened. **B** The most positively correlated immunocyte-m6A regulators pair in MG. **C** The most negatively correlated immunocyte-m6A regulators in MG

**Fig. 5:**
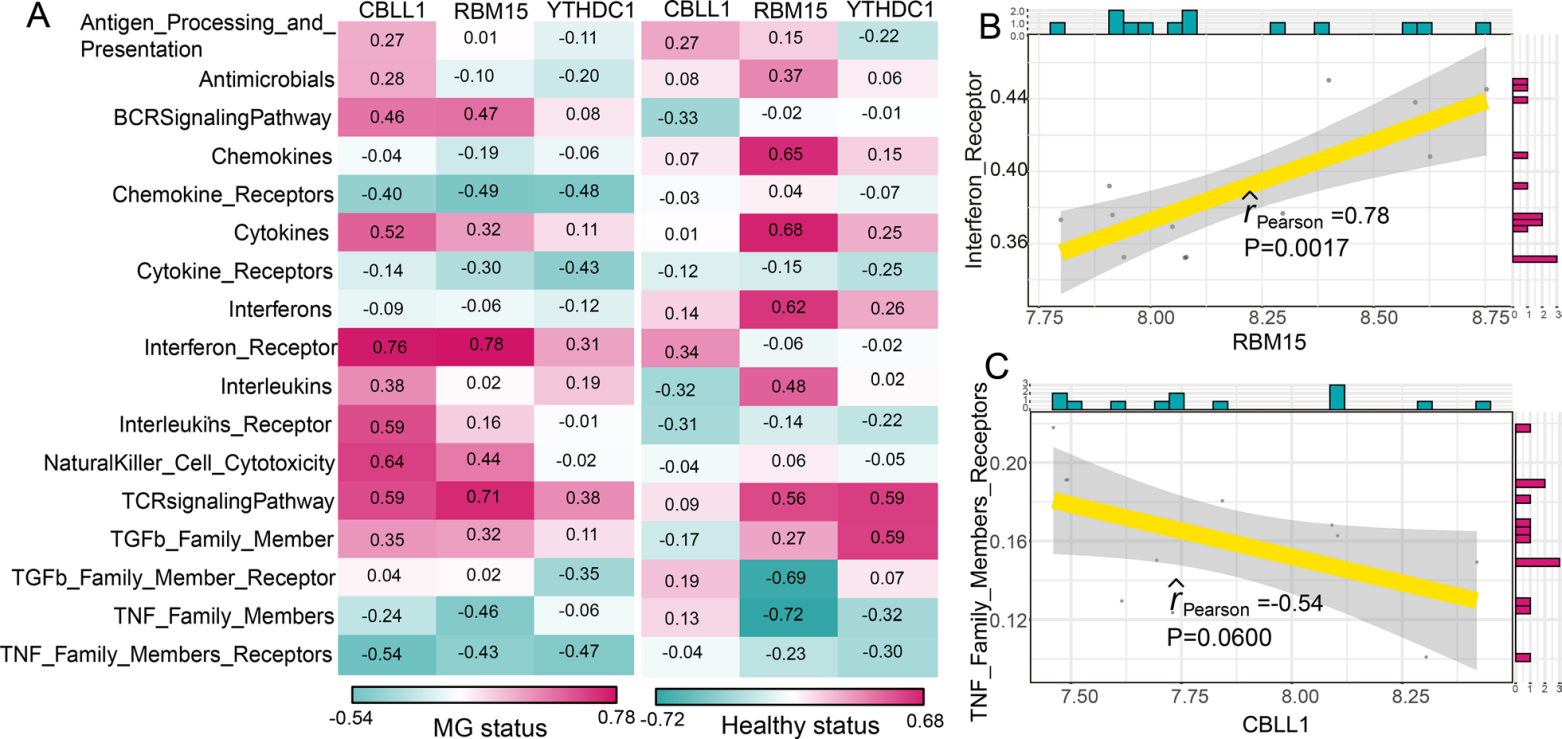
The correlations between immune reaction pathways and dysregulated m6A regulators. **A** The dot-plot showed the correlations between each immune reaction pathway and each dysregulated m6A regulators in MG samples and healthy samples. The positive correlation became stronger as the red color darkened, and the negative correlation became stronger as the green color darkened. **B** The most positively correlated immune reaction pathway-m6A regulators pair in MG. **C** The most negatively correlated immune reaction pathway-m6A regulators in MG

**Fig. 6:**
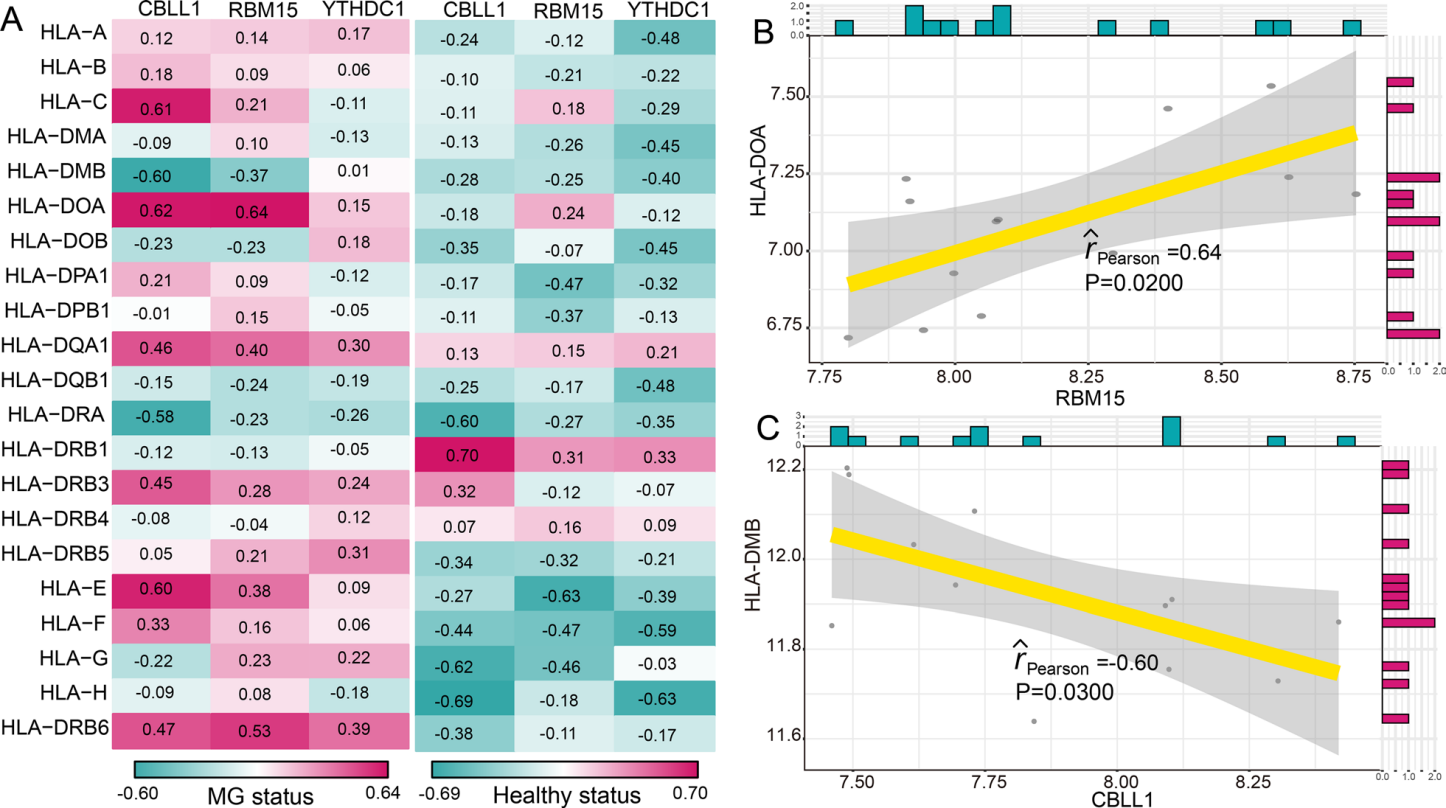
The correlations between HLA genes and dysregulated m6A regulators. **A** The dot-plot showed the correlations between each HLA gene and each dysregulated m6A regulators in MG samples and healthy samples. The positive correlation became stronger as the red color darkened, and the negative correlation became stronger as the green color darkened. **B** The most positively correlated HLA-m6A regulators pair in MG. **C** The most negatively correlated HLA-m6A regulators in MG

As we known, the pathogenesis of MG is closely related to immunity. Thus, we investigated the relationships between m6A modification regulators and immune characteristics including immune cell subpopulations, immune reaction pathways and HLA gene expression according to a previous study [21]. We found that the correlations between m6A regulators and immune cell subpopulations have changed between healthy status and MG status (Fig. 4A). The most positively correlated m6A regulator-immune cell pair was RBM15-CD56dim natural killer cell, which means a higher expression of RBM15 and a higher score of CD56dim natural killer cell were found in MG (Fig. 4B). While the most negatively correlated pair was RBM15-T follicular helper cell, suggesting a higher RBM15 expression and a lower score of T follicular helper cell in MG (Fig. 4C). Similarly, the changes of correlations between immune reaction pathways and m6A regulators were also observed from healthy status to MG patients (Fig. 5A). RBM15-Interferon Receptor was the most positively correlated pair, and CBLL1-TNF Family Members Receptors was the most negatively correlated pair (Fig. 5B and 5C). However, the correlation between CBLL1 and TNF Family Members Receptors was not significant (P = 0.06). The results indicated that RBM15 might play a crucial important role in immune reaction pathway of Interferon Receptor. As for the correlations of HLA genes and m6A regulators, a dramatically change occurred between healthy and MG samples (Fig. 6A). Many m6A regulators-HLA gene pairs that were negatively correlated became positively correlated or positively correlated became negatively correlated. Where the most positively correlated m6A regulators-HLA pair was RBM15-HLA-DOA (Fig. 6B), and the most negatively correlated was CBLL1-HLA-DMB (Fig. 6C).

### Different m6A RNA methylation modification patterns in MG

**Fig. 7:**
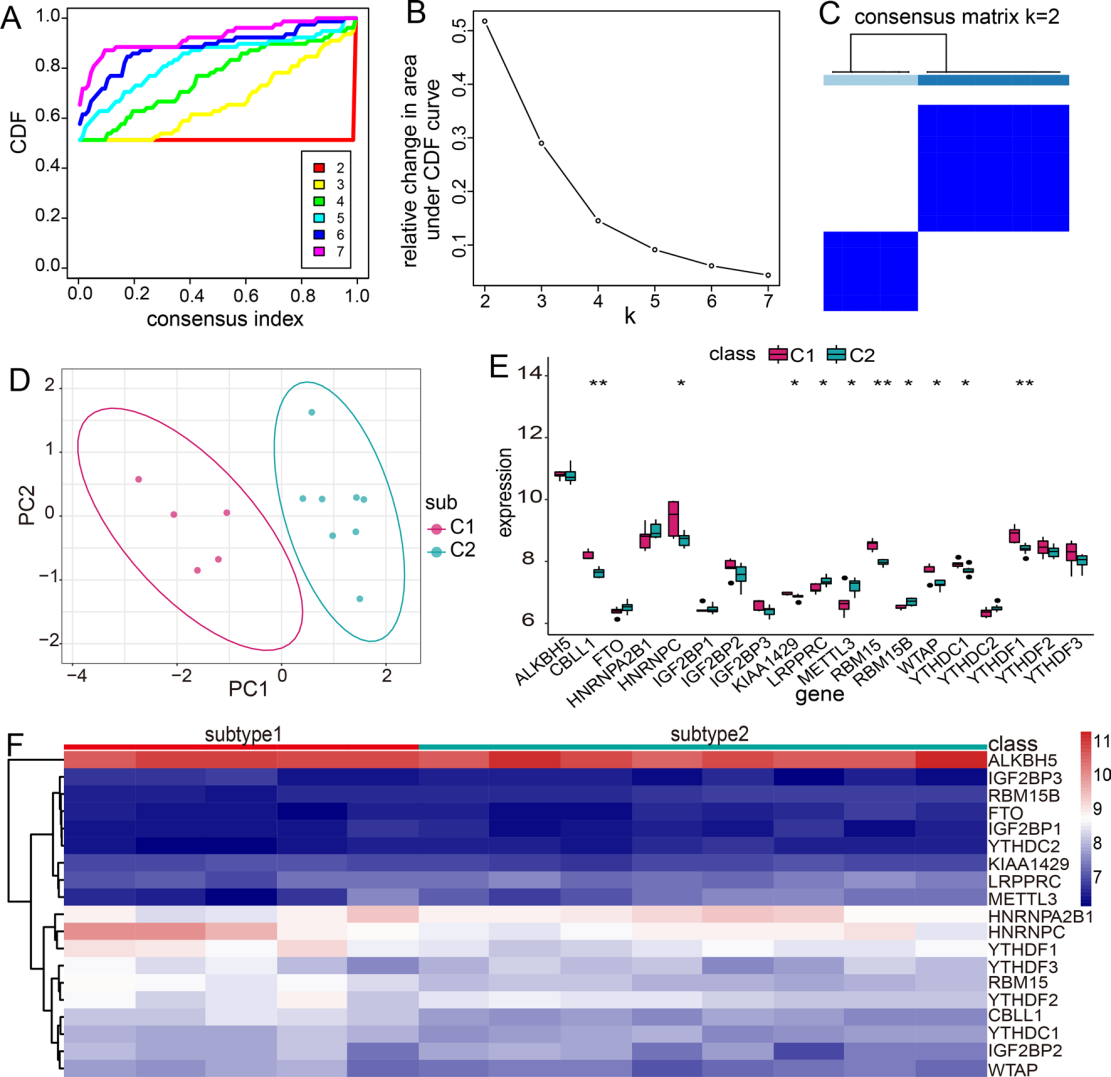
Identification of distinct m6A modification patterns by unsupervised consensus clustering. **A** Consensus clustering cumulative distribution function (CDF) for k = 2–7. **B** Relative change in area under CDF curve for k = 2–7. **C** Heatmap of the matrix of co-occurrence proportions for MG samples. **D** PCA analysis for the transcriptome profiles of the two different modification subtypes. **E**, **F** The expression status of all m6A regulators in the two subtypes

m6A RNA methylation can influence gene expression through regulating RNA stability, RNA structure, and alternative splicing [22, 23]. Thus, we performed consensus clustering for MG with the dysregulated m6A regulators in MG utilizing the Consensus Cluster Plus R package. As a result, 13 MG samples were clustered into two subtypes, including subtype-1 (n = 5) and subtype-2 (n = 8) (Fig. 7A-C). Furtherly, PCA analysis of these two subtypes revealed that there was a significantly difference in transcriptome expression between the two modification patterns (Fig. 7D). To explore the relationship between 19 m6A RNA methylation regulators and two subtypes, we compared the expression levels of all m6A regulators in two modification patterns (Fig. 7E, F). We found that CBLL1, HNRNPC, KIAA1429, RBM15, WTAP, YTHDC1, and YTHDF1 were mostly upregulated in subtype-1. LRPPRC, METTL3, and RBM15B were upregulated in subtype-2.

### Immune characteristics in the two distinct m6A modification patterns

**Fig. 8:**
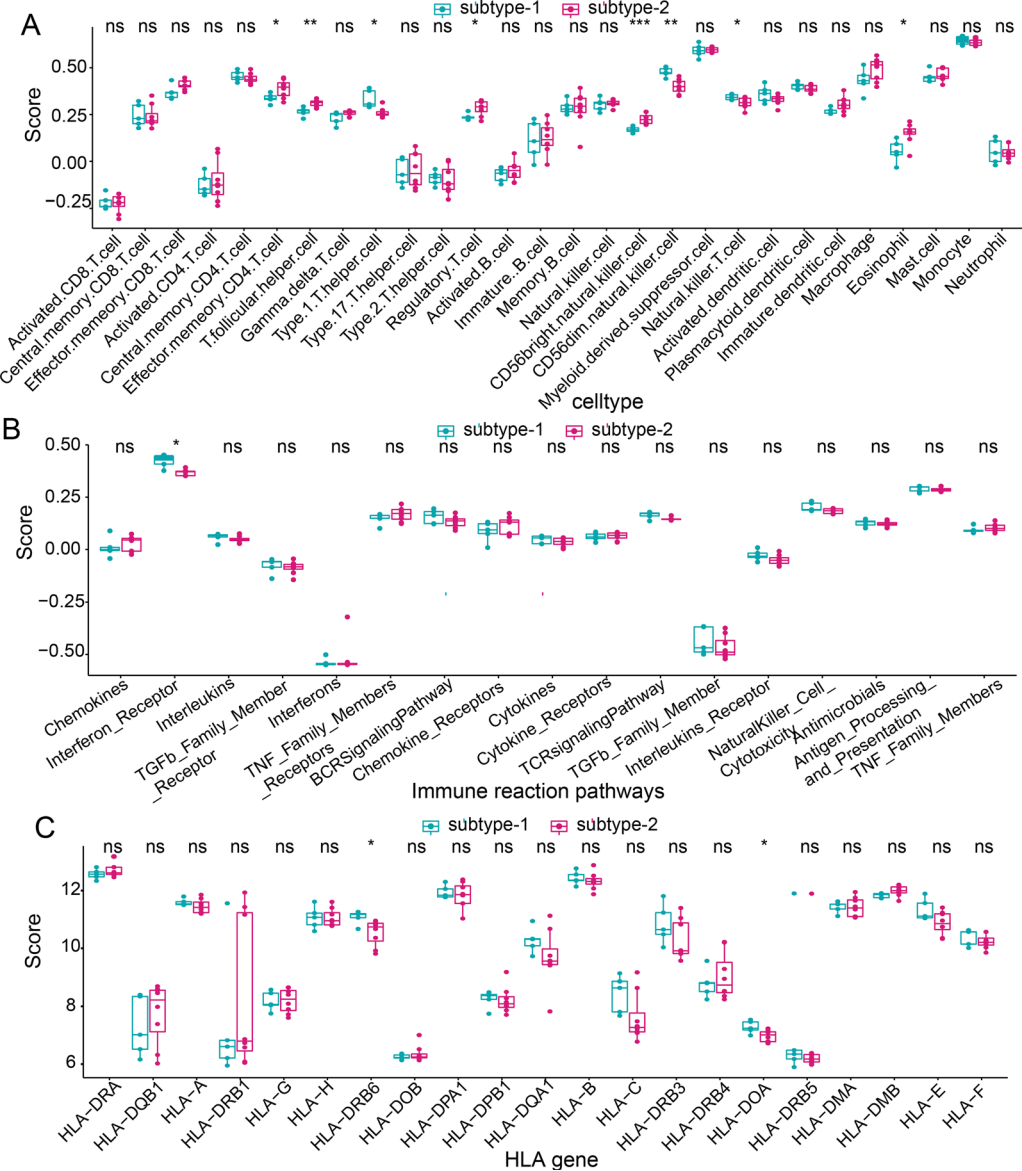
Different immune characteristics in two distinct m6A modification subtypes. **A** The abundance differences of each immune immunocyte in two modification subtypes. **B** The activity differences of each immune reaction pathway in two modification subtypes. **C** The expression differences of each HLA gene in two modification subtypes

To portray the immune characteristics of two different m6A modification patterns, the expression status of immune cell subpopulations, immune reaction pathways and HLA gene expression was compared. As expected, different immune features were observed between two subtypes. For example, higher level of Gamma delta T cell, CD56bright natural killer cell, and Myeloid derived suppressor cell were enriched in subtype-1, while higher levels of Central memory CD4 T cell, Effector memory CD4 T cell, Type 2T helper cell, Natural killer cell and Macrophage were enriched subtype-2 (Fig. 8A). As for immune reaction pathway and HLA genes, there were only slight differences between the two modification patterns. Just more active Interferon Receptor (Fig. 8B) and higher expression level of HLA-DRB6 and HLA-DOA (Fig. 8C) were observed in subtype-1.

### Identification of biological functions and modules related to two m6A modification patterns

**Fig. 9:**
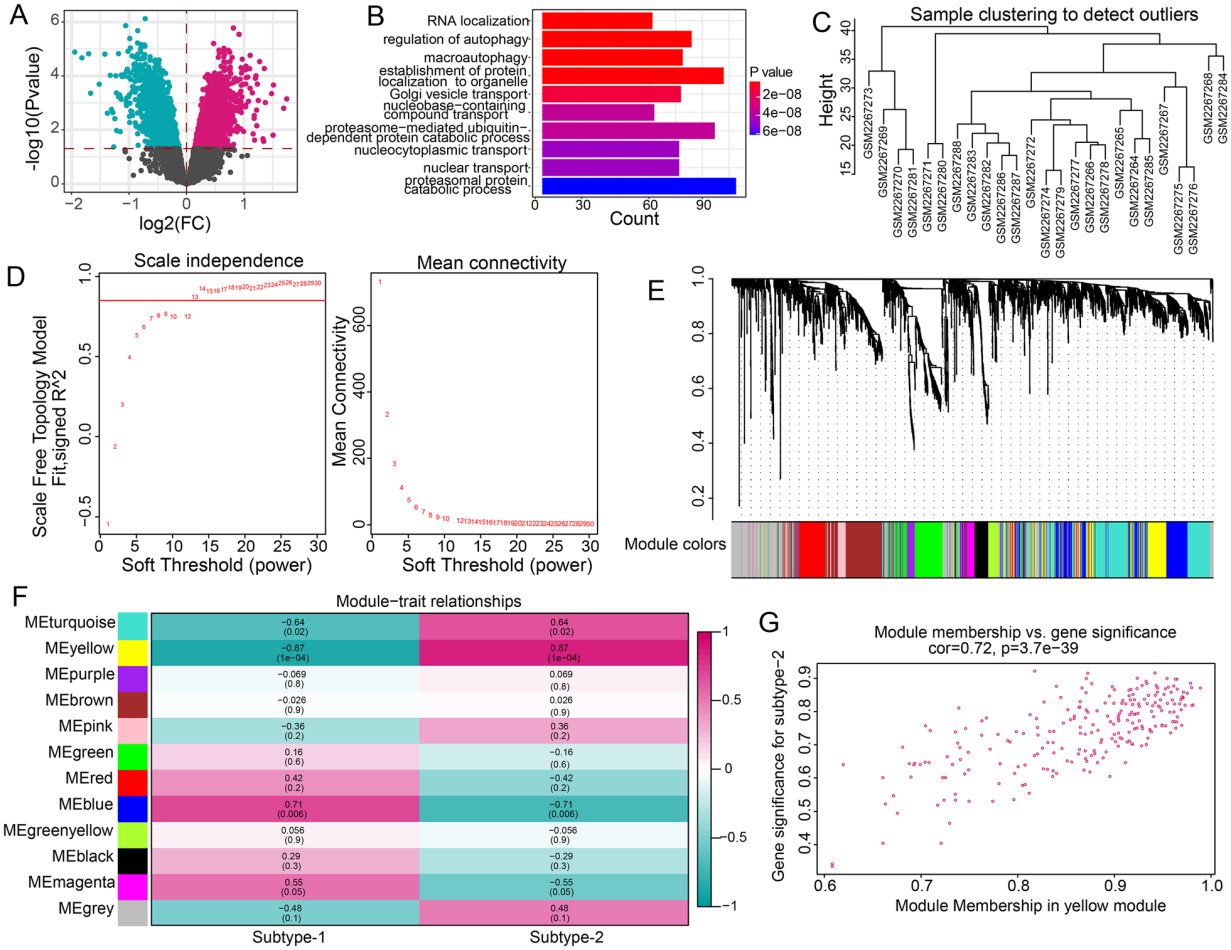
The underlying biological function characteristics of different m6A modification subtypes. **A** The differentially expressed genes between two subtypes. **B** The top 10 of GO-BP enrichment analysis of the above differentially expressed genes. **C** The sample clustering was performed according to the expression data of all samples. **D** Analysis of the scale free ft index and analysis of the mean connectivity for various soft-thresholding powers. **E** Gene dendrogram obtained by average linkage hierarchical clustering. The color row underneath the dendrogram shows the module assignment determined by the Dynamic Tree Cut, in which 12 modules were identified. **F** The heatmap showed the correlation between module eigengenes and the two modification patterns. **G** A scatterplot of gene significance (GS) for subtype-2 versus module membership (MM) in the yellow module. GS and MM exhibit a very significant correlation, implying that hub genes of the yellow module also tend to be highly correlated with subtype-2

To further understand the potential biological functions of two m6A modification patterns, we determined 3029 differentially expressed genes between two m6A modification patterns (Fig. 9A). Then, GO-BP enrichment analysis of the above genes was performed and the results uncovered that the mainly enriched GO-BP terms included RNA localization, regulation of autophagy, and macroautophagy, et al. (Fig. 9B). Finally, a comprehensive gene landscape was constructed and gene–gene modules were identified using WGCNA (Fig. 9C–E). We obtained 12 gene modules and then analyzed the relationships between two m6A modification patterns and modules (Fig. 9F). We found that yellow module was the most positively correlated to subtype-2 and the genes in yellow module was closely associated with subtype-2 (Fig. 9G).

## Discussion

MG is a neurological autoantibody-mediated disorder, involving T cells, B cells, and complements. In recent years, with the improvement of diagnostic techniques and the prolongation of the average survival time of MG patients, the incidence and prevalence of MG are significantly increasing. However, due to the complex pathogenesis and heterogeneity of MG, no one treatment therapy is best for all MG patients [24]. To data, there is short of effective biomarkers of treatment and prognosis for MG worldwide. Therefore, the exact molecular mechanism of MG urgently needs to be clearly understood, thereby identifying precise biomarkers for MG. m6A RNA methylation modification, a new epigenetic pattern of gene expression regulation, has attracted extensive attention among the research community. The process of m6A modification is regulated by methyltransferases, demethylases, and binding proteins. The indispensable roles of m6A modification in both innate and adaptive immune reactions have been reported [25]. Recently, researchers have discovered that m6A modification exhibits a great importance in some autoimmune diseases [26]. However, few studies have focused on m6A in MG. To the best of our knowledge, this work was the first systematic bioinformatics analysis of the landscapes of m6A modification regulators in MG field, which would provide a reliable direction for future-specific experimental design of MG.

In this study, we first found that two “writers” (CBLL1 and RBM15) and one “reader” (YTHDF1) presented statistical significance between MG and healthy samples, suggesting their possible functional importance in the pathogenesis of MG. Generally, methyltransferases are responsible for the formation of m6A modification [27]. A study has confirmed that E3 ubiquitin ligase CBLL1 possessed the potential m6A catalytic activity [28]. RBM15 and its homologue RBM15B, additional components of the methyltransferase complex, interact with METTL3 in a WTAP-dependent manner and the absence of RBM15/RBM15B cause significant reductions of m6A in mRNA [28, 29]. As a member of YTH protein family, YTHDF1 is the most versatile and powerful reader protein of m6A modification, which can regulate gene expression through different mechanisms [30]. Some studies have reported that CBLL1, RBM15, and YTHDF1 were associated with immune processes and participated in the development of multiple diseases. For example, Zheng et al. found that higher CBLL1 expression was associated with a better prognosis in breast cancer than lower CBLL1 expression, and the results of functional analysis showed that CBLL1 was related to immune-related pathway [31]. Another study showed that the expression of RBM15 was positively correlated with immune infiltrating cells in pancreatic adenocarcinoma, and RBM15 knockdown suppressed the proliferation of pancreatic cancer cells [32]. Tsuchiya et al. discovered that knockdown of YTHDF1 in lung cancer cells upregulated tumor PD-L1 expression and altered multiple immune-related genes in vitro, and high expressions of YTHDF1 was associated with a favorable prognostic outcome of lung cancer patients and downregulation of PD-L1 [33]. To further explore the roles and the downstream molecular mechanisms of m6A regulators in MG, we performed correlation analysis and function enrichment analysis. We found that the correlations among CBLL1, RBM15, and YTHDF1 became stronger in MG than healthy samples, which indicated they might be involved in the occurrence of MG, together. According to the common genes targeted by CBLL1, RBM15, and YTHDF1, the most enriched GO-BP term was histone modification and the most significant pathway was Wnt signaling pathway. As we all know, Agrin-LRP4-MuSK signaling pathway is the primary mechanism for the formation of neuromuscular junction and the most important pathway for AChR clustering [34, 35]. While the Wnt signaling pathway and the Agrin-LRP4-MuSK signaling pathway engaged in crosstalk. Dvl1 was a central effector of all the canonical and non-canonical Wnt signaling pathway, which could interact with MuSK to regulate AChR clustering [36, 37]. These findings suggested that CBLL1, RBM15, and YTHDF1 might participate in the development of MG through regulating Wnt signaling pathway.

Jin et al. identified 13 major cell clusters and 39 subgroups of cells between new-onset myasthenia gravis and healthy controls based on single-cell sequencing [38]. They further found that CD180- B cell subgroups in MG were associated with disease activity and the anti-AChR antibody [38]. This study suggested some immune cell subpopulations might play a crucial role in the pathogenesis of MG. Therefore, we compared the correlations of three MG-related m6A regulators and immune cell subpopulations between MG samples and healthy samples. The results showed that the most positively correlated m6A regulator-immune cell subpopulation pair was RBM15-CD56dim natural killer cell. In our study, RBM15 is upregulated in MG, while a previous study confirmed that the frequencies of peripheral CD56dim natural killer cell in patients with new-onset MG and stable MG were higher than those in healthy controls [39], which provided support for our result. In addition, Interferon Receptor and HLA-DOA were the most positively correlated immune reaction pathway and HLA gene with RBM15, respectively. These results demonstrated that RBM15 might exert an important impact on the immune mechanism of MG. Considering the heterogeneity of MG, we performed consensus clustering for MG using dysregulated m6A regulators to identify different modification patterns. As a result, two m6A modification related subtypes were obtained. We found that CBLL1, HNRNPC, KIAA1429, RBM15, WTAP, YTHDC1, and YTHDF1 were overexpressed in subtype-1, whereas LRPPRC, METTL3, and RBM15B were overexpressed in subtype-2. The immune characteristics between two subtypes also exhibited different enrichment status, such as higher level of Gamma delta T cell, CD56bright natural killer cell, and Myeloid derived suppressor cell in subtype-1, while higher level of Type 2 T helper cell, Natural killer cell and Macrophage in subtype-2, as well as more active Interferon Receptor and higher expression level of HLA-DRB6 and HLA-DOA in subtype-1. GO-BP enrichment analysis indicated that differentially expressed genes between two subtypes were mainly enriched in RNA localization, regulation of autophagy, and macroautophagy. Furtherly, the results of WGCNA analysis showed that yellow module was the most positively correlated to subtype-2 and was the most negatively correlated to subtype-1. Taken together, distinct m6A modification patterns indeed had different molecular characteristics. This classification strategy for subtypes contributes to explain the heterogeneity of disease and understand the potential molecular mechanism of MG, and can help make a better individualized treatment plan for MG patients.

## Conclusion

In conclusion, we for the first time investigated the expression features and biological functions of m6A regulators in MG, along with relationships between MG-related m6A regulators and immune characteristics. Our results hinted that m6A regulators might play important roles in the pathogenesis of MG. We further draw a diagram to demonstrate how m6A regulators exert the potential regulatory roles in the mechanism of MG (Additional file 6: Figure S1). As shown, dysregulated m6A regulators might be involved in the development of MG through interplaying with some important signaling pathways or immune cells. The findings of our work will offer a theoretical basis for further designing experiment to research the roles of m6A modification in MG and develop a novel direction for researchers to explore the pathogenesis of other diseases, especially autoimmune diseases. However, our study also exists limitations. First, this work is based on bioinformatics analysis, and many findings are theoretically valid, the accuracy of which remains to be verified by designing wet experiments. Second, MG sample included in this study is small, and it is necessary to expand the samples for further research. Nevertheless, our study did reveal the significant effects m6A modification has on the molecular mechanism of MG and provided a novel perspective for understanding the potential pathogenesis of MG.

## Supplementary Information

**Additional file 1: Table S1.** The detailed clinical information of MG group.

**Additional file 2: Table S2.** Targets of CBLL1, RBM15, and YTHDF1 from m6A2Target database.

**Additional file 3: Table S3.** Targets modified by CBLL1, RBM15, and YTHDF1 in MG (|r|>0.5, P value<0.05).

**Additional file 4: Table S4.** GO-BP terms enriched by targets of CBLL1, RBM15, and YTHDF1, respectively.

**Additional file 5: Table S5.** Pathways enriched by targets of CBLL1, RBM15, and YTHDF1, respectively.

**Additional file 6: Figure S1.** A diagram illustrating the potential mechanism mediated by dysregulated m6A regulators in MG.

## Data Availability

Although the study is based on mined data (GEO), the analysis and plots are original. The datasets used and/or analysed during the current study are available from the corresponding author on reasonable request.

## References

[1] Gomez AM, Van Den Broeck J, Vrolix K, Janssen SP, Lemmens MA, Van Der Esch E (2010). Antibody effector mechanisms in myasthenia gravis-pathogenesis at the neuromuscular junction. Autoimmunity.

[2] Huijbers MG, Marx A, Plomp JJ, Le Panse R, Phillips WD (2022). Advances in the understanding of disease mechanisms of autoimmune neuromuscular junction disorders. Lancet Neurol.

[3] Punga AR, Maddison P, Heckmann JM, Guptill JT, Evoli A (2022). Epidemiology, diagnostics, and biomarkers of autoimmune neuromuscular junction disorders. Lancet Neurol.

[4] Çebi M, Durmus H, Aysal F, Özkan B, Gül GE, Çakar A (2020). CD4(+) T cells of myasthenia gravis patients are characterized by increased IL-21, IL-4, and IL-17A productions and higher presence of PD-1 and ICOS. Front Immunol.

[5] Zhang X, Liu S, Chang T, Xu J, Zhang C, Tian F (2016). Intrathymic Tfh/B cells interaction leads to ectopic GCs formation and anti-AChR antibody production: central role in triggering MG occurrence. Mol Neurobiol.

[6] Mathoux J, Henshall DC, Brennan GP (2021). Regulatory mechanisms of the RNA modification m(6)A and significance in brain function in health and disease. Front Cell Neurosci.

[7] Zhu ZM, Huo FC, Pei DS (2020). Function and evolution of RNA N6-methyladenosine modification. Int J Biol Sci.

[8] Cao G, Li HB, Yin Z, Flavell RA (2016). Recent advances in dynamic m6A RNA modification. Open Biol.

[9] Wang H, Hu X, Huang M, Liu J, Gu Y, Ma L (2019). Mettl3-mediated mRNA m(6)A methylation promotes dendritic cell activation. Nat Commun.

[10] Zhou J, Zhang X, Hu J, Qu R, Yu Z, Xu H (2021). m(6)A demethylase ALKBH5 controls CD4(+) T cell pathogenicity and promotes autoimmunity. Sci Adv.

[11] Luo Q, Rao J, Zhang L, Fu B, Guo Y, Huang Z (2020). The study of METTL14, ALKBH5, and YTHDF2 in peripheral blood mononuclear cells from systemic lupus erythematosus. Mol Genet Genomic Med.

[12] Mo XB, Lei SF, Qian QY, Guo YF, Zhang YH, Zhang H (2019). Integrative analysis revealed potential causal genetic and epigenetic factors for multiple sclerosis. J Neurol.

[13] Shi W, Zheng Y, Luo S, Li X, Zhang Y, Meng X (2021). METTL3 promotes activation and inflammation of FLSs through the NF-κB signaling pathway in rheumatoid arthritis. Front Med.

[14] Song RH, Zhao J, Gao CQ, Qin Q, Zhang JA (2021). Inclusion of ALKBH5 as a candidate gene for the susceptibility of autoimmune thyroid disease. Adv Med Sci.

[15] Mamrut S, Avidan N, Truffault F, Staun-Ram E, Sharshar T, Eymard B (2017). Methylome and transcriptome profiling in Myasthenia Gravis monozygotic twins. J Autoimmun.

[16] Deng S, Zhang H, Zhu K, Li X, Ye Y, Li R (2021). M6A2Target: a comprehensive database for targets of m6A writers, erasers and readers. Brief Bioinform.

[17] Shen S, Wang G, Zhang R, Zhao Y, Yu H, Wei Y (2019). Development and validation of an immune gene-set based Prognostic signature in ovarian cancer. EBioMedicine.

[18] Bhattacharya S, Andorf S, Gomes L, Dunn P, Schaefer H, Pontius J (2014). ImmPort: disseminating data to the public for the future of immunology. Immunol Res.

[19] Chai RC, Wu F, Wang QX, Zhang S, Zhang KN, Liu YQ (2019). m(6)A RNA methylation regulators contribute to malignant progression and have clinical prognostic impact in gliomas. Aging.

[20] Langfelder P, Horvath S (2008). WGCNA: an R package for weighted correlation network analysis. BMC Bioinform.

[21] Zhang X, Zhang S, Yan X, Shan Y, Liu L, Zhou J (2021). m6A regulator-mediated RNA methylation modification patterns are involved in immune microenvironment regulation of periodontitis. J Cell Mol Med.

[22] Wang X, Lu Z, Gomez A, Hon GC, Yue Y, Han D (2014). N6-methyladenosine-dependent regulation of messenger RNA stability. Nature.

[23] Liu N, Zhou KI, Parisien M, Dai Q, Diatchenko L, Pan T (2017). N6-methyladenosine alters RNA structure to regulate binding of a low-complexity protein. Nucleic Acids Res.

[24] Sanders DB, Wolfe GI, Benatar M, Evoli A, Gilhus NE, Illa I (2016). International consensus guidance for management of myasthenia gravis: executive summary. Neurology.

[25] Zheng Q, Hou J, Zhou Y, Li Z, Cao X (2017). The RNA helicase DDX46 inhibits innate immunity by entrapping m(6)A-demethylated antiviral transcripts in the nucleus. Nat Immunol.

[26] Wang Y, Li L, Li J, Zhao B, Huang G, Li X (2021). The emerging role of m6A modification in regulating the immune system and autoimmune diseases. Front Cell Dev Biol.

[27] Liu C, Yang Z, Li R, Wu Y, Chi M, Gao S (2021). Potential roles of N6-methyladenosine (m6A) in immune cells. J Transl Med.

[28] Horiuchi K, Kawamura T, Iwanari H, Ohashi R, Naito M, Kodama T (2013). Identification of Wilms' tumor 1-associating protein complex and its role in alternative splicing and the cell cycle. J Biol Chem.

[29] Patil DP, Chen CK, Pickering BF, Chow A, Jackson C, Guttman M (2016). m(6)A RNA methylation promotes XIST-mediated transcriptional repression. Nature.

[30] Chen Z, Zhong X, Xia M, Zhong J (2021). The roles and mechanisms of the m6A reader protein YTHDF1 in tumor biology and human diseases. Mol Ther Nucleic Acids.

[31] Zhang B, Gu Y, Jiang G (2020). Expression and prognostic characteristics of m(6) A RNA methylation regulators in breast cancer. Front Genet.

[32] Zhao Z, Ju Q, Ji J, Li Y, Zhao Y (2022). N6-methyladenosine methylation regulator RBM15 is a potential prognostic biomarker and promotes cell proliferation in pancreatic adenocarcinoma. Front Mol Biosci.

[33] Tsuchiya K, Yoshimura K, Inoue Y, Iwashita Y, Yamada H, Kawase A (2021). YTHDF1 and YTHDF2 are associated with better patient survival and an inflamed tumor-immune microenvironment in non-small-cell lung cancer. Oncoimmunology.

[34] Fish LA, Fallon JR (2020). Multiple MuSK signaling pathways and the aging neuromuscular junction. Neurosci Lett.

[35] Ohkawara B, Ito M, Ohno K (2021). Secreted signaling molecules at the neuromuscular junction in physiology and pathology. Int J Mol Sci.

[36] Luo ZG, Wang Q, Zhou JZ, Wang J, Luo Z, Liu M (2002). Regulation of AChR clustering by Dishevelled interacting with MuSK and PAK1. Neuron.

[37] Henriquez JP, Webb A, Bence M, Bildsoe H, Sahores M, Hughes SM (2008). Wnt signaling promotes AChR aggregation at the neuromuscular synapse in collaboration with agrin. Proc Natl Acad Sci USA.

[38] Jin W, Yang Q, Peng Y, Yan C, Li Y, Luo Z (2021). Single-cell RNA-Seq reveals transcriptional heterogeneity and immune subtypes associated with disease activity in human myasthenia gravis. Cell Discov.

[39] Liu RT, Li W, Guo D, Yang CL, Ding J, Xu JX (2021). Natural killer cells promote the differentiation of follicular helper T cells instead of inducing apoptosis in myasthenia gravis. Int Immunopharmacol.

