# Supplementary figures and images for: Landscape analysis of m6A modification regulators related biological functions and immune characteristics in myasthenia gravis
Source: J Transl Med. 2023 Mar 2;21:166. doi: 10.1186/s12967-023-03947-5 (PMC9983271; doi:10.1186/s12967-023-03947-5)

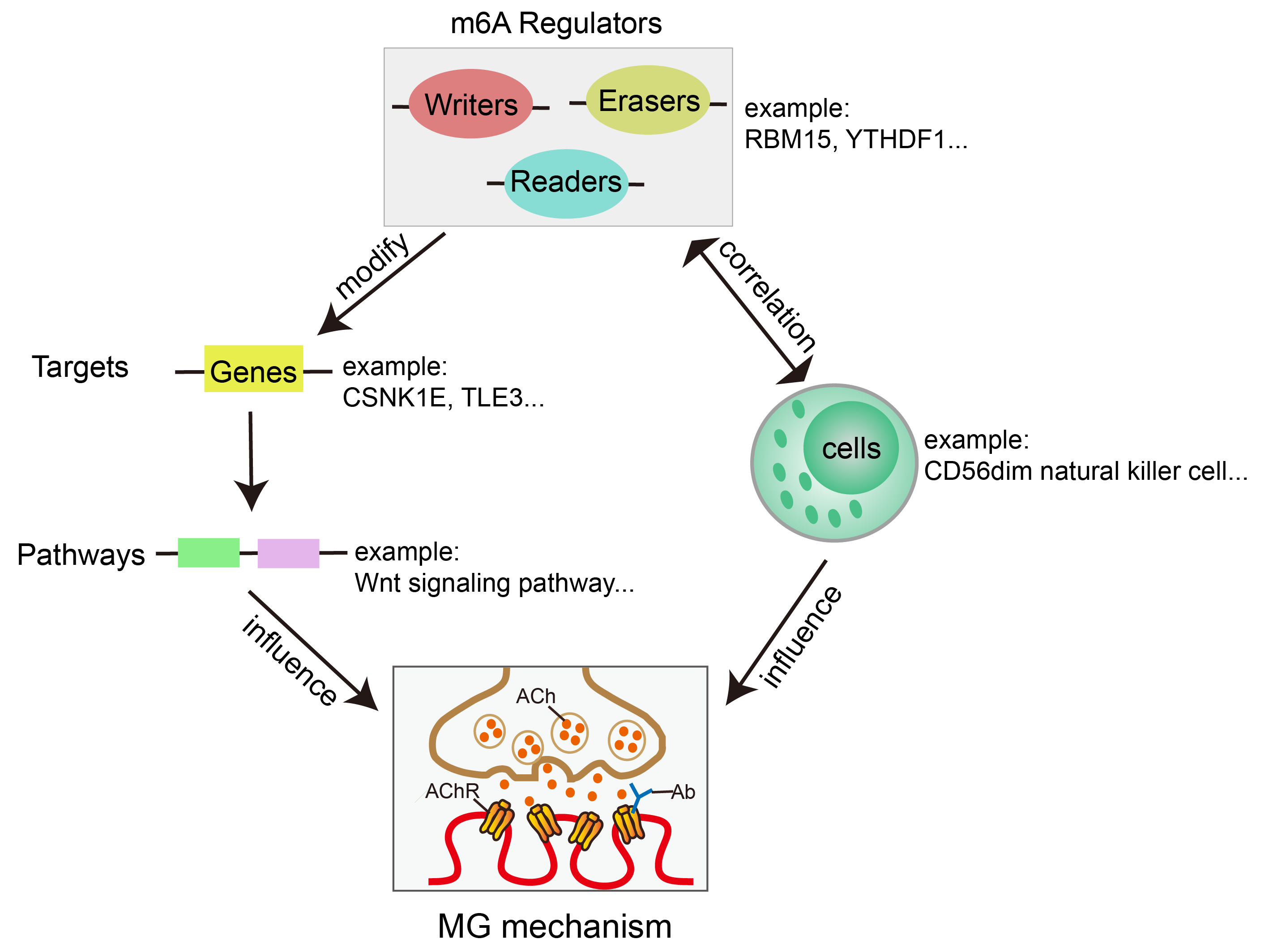

Supplement: Supplementary file 6 — Additional file 6: Figure S1. A diagram illustrating the potential mechanism mediated by dysregulated m6A regulators in MG. [file 12967_2023_3947_MOESM6_ESM.tif]
